# Understanding P-Rex regulation: structural breakthroughs and emerging perspectives

**DOI:** 10.1042/BST20231546

**Published:** 2024-07-18

**Authors:** Gareth D. Jones, Andrew M. Ellisdon

**Affiliations:** Cancer Program, Biomedicine Discovery Institute, Monash University, Clayton 3800, Victoria, Australia

**Keywords:** G-proteins, GTPases, guanine nucleotide exchange factor, phosphatidylinositol, Rac, structural biology

## Abstract

Rho GTPases are a family of highly conserved G proteins that regulate numerous cellular processes, including cytoskeleton organisation, migration, and proliferation. The 20 canonical Rho GTPases are regulated by ∼85 guanine nucleotide exchange factors (GEFs), with the largest family being the 71 Diffuse B-cell Lymphoma (Dbl) GEFs. Dbl GEFs promote GTPase activity through the highly conserved Dbl homology domain. The specificity of GEF activity, and consequently GTPase activity, lies in the regulation and structures of the GEFs themselves. Dbl GEFs contain various accessory domains that regulate GEF activity by controlling subcellular localisation, protein interactions, and often autoinhibition. This review focuses on the two phosphatidylinositol (3,4,5)-trisphosphate (PI(3,4,5)P_3_)-dependent Rac exchangers (P-Rex), particularly the structural basis of P-Rex1 autoinhibition and synergistic activation. First, we discuss structures that highlight the conservation of P-Rex catalytic and phosphoinositide binding activities. We then explore recent breakthroughs in uncovering the structural basis for P-Rex1 autoinhibition and detail the proposed minimal two-step model of how PI(3,4,5)P_3_ and Gβγ synergistically activate P-Rex1 at the membrane. Additionally, we discuss the further layers of P-Rex regulation provided by phosphorylation and P-Rex2-PTEN coinhibitory complex formation, although these mechanisms remain incompletely understood. Finally, we leverage the available data to infer how cancer-associated mutations in P-Rex2 destabilise autoinhibition and evade PTEN coinhibitory complex formation, leading to increased P-Rex2 GEF activity and driving cancer progression and metastasis.

## Background

**Figure 1.**
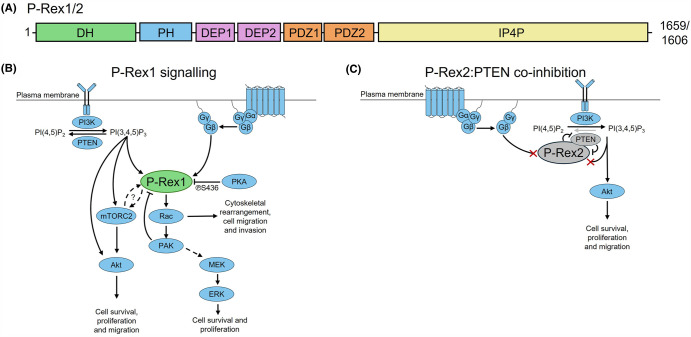
P-Rex GEF domain organisation and signalling. (**A**) Domain organisation of P-Rex1 and P-Rex2. (**B**) Schematic of P-Rex1 signalling summarising the synergistic activation of P-Rex1 by PI(3,4,5)P_3_ and Gβγ. The dashed lines between P-Rex and mTORC2 indicate that the functional consequences of this interaction require further investigation. The dashed line between PAK and MEK indicates that in some contexts P-Rex is known to be dispensable for MAPK signalling [4]. (**C**) Consequences of P-Rex2:PTEN co-inhibitory complex formation on P-Rex2 and PTEN signalling pathways.

The phosphatidylinositol (3,4,5)-trisphosphate (PI(3,4,5)P_3_)-dependent Rac exchangers (P-Rex) family consists of two large Diffuse B-cell Lymphoma (Dbl) guanine nucleotide exchange factors (GEFs) for Rac type Rho GTPases [1–3]. P-Rex1 and P-Rex2 share a comparable seven-domain arrangement with ∼60% overall sequence identity (Figure 1A). The catalytic core of the P-Rex proteins is the N-terminal Dbl homology (DH) domain that activates target Rho GTPases by catalysing nucleotide exchange from the inactive GDP-bound state to the active GTP-bound conformation [5]. The DH domain is followed by a pleckstrin homology (PH) domain, forming a tandem domain arrangement typical of Dbl GEFs with the DH domain encoding GEF activity and the PH domain regulating membrane recruitment through phosphoinositide binding [5,6]. C-terminal to the DH-PH domains are two Dishevelled EGL10 and pleckstrin (DEP) domains, two postsynaptic density 95, discs large and zonula occludens 1 (PDZ) domains, and a large C-terminal inositol polyphosphate 4-phosphatase (IP4P) domain [1]. Notably, although the IP4P domain contains a canonical dual specificity phosphatase active site motif (CX_5_R), no phosphatase activity has been detected to date [1,7,8]. Additionally, a shorter splice variant of P-Rex2, P-Rex2b, has been identified that terminates within the IP4P domain. This variant is 980 amino acids long and terminates before the CX_5_R motif of the IP4P domain [3].

The P-Rex family are synergistically activated at the plasma membrane (Figure 1B). Under basal conditions, P-Rex proteins are primarily cytosolic and maintained in an autoinhibited conformation with highly reduced GEF activity [1,5,6]. Canonically, activation is achieved by binding PI(3,4,5)P_3_, generated by phosphoinositide 3-kinase (PI3K) activation, and the heterodimeric Gβγ G protein complex, which is released downstream of G protein-coupled receptor (GPCR) stimulation [1–3,5]. The requirement for both PI(3,4,5)P_3_ and Gβγ binding for efficient P-Rex activation is unique amongst GEFs, allowing the P-Rex proteins to integrate cellular signals downstream of class 1 PI3Ks and GPCRs [9]. Adding a further layer of complexity, P-Rex2, but not P-Rex1, forms a complex with the tumour suppressor protein phosphatase and tensin homolog (PTEN), resulting in the co-inhibition of both proteins (Figure 1C) [10–12].

Increased P-Rex activity has been implicated in several cancers, with P-Rex1 typically dysregulated through up-regulated expression levels and P-Rex2 through direct mutation [13]. Within the cell, P-Rex1 catalysed Rac1 promotes cytoskeletal rearrangement, cell survival, migration, and invasion both directly and via the activation of the PAK and RAF/MEK/ERK signalling cascade [1,14–19]. The cross-talk between PAK and the MAPK signalling cascade is cell line dependent. While P-Rex1 is dispensable for ERK activation in breast cancer cell lines [4,14], in acute myeloid leukaemia-derived cell lines, P-Rex1 is required for MAPK signalling [20]. Independently of GTPase activation, P-Rex2 binds and inhibits PTEN, preventing the conversion of PI(3,4,5)P_3_ to PI(4,5)P_2_, and resulting in increased Akt activation and the promotion of cell survival, proliferation and migration [10,11]. In contrast, P-Rex1 does not bind PTEN [21], and has no reported effect on Akt upstream of Rac1 activation [4]. Lastly, P-Rex1 and P-Rex2 interact with the nutrient-sensing hub mechanistic target of rapamycin (mTOR) kinase via their DEP domains, with mTORC2-bound P-Rex1 potentially driving Rac activation and cell migration [22]. However, the functional consequences of the P-Rex:mTOR interaction are not yet well understood and require further investigation.

## P-Rex catalytic activity and phosphoinositide binding

How a single seven-domain protein fold co-ordinates autoinhibition under basal conditions and synergistic activation by PI(3,4,5)P_3_ and Gβγ binding has propelled numerous structural investigations of P-Rex1 and P-Rex2. The involvement of P-Rex proteins in cancer enhances the significance of investigating these unique protein structures by revealing avenues for therapeutic targeting and deepening our understanding of how cancer-associated mutations in P-Rex2 dysregulate its activity to drive cancer progression and metastasis.

**Figure 2.**
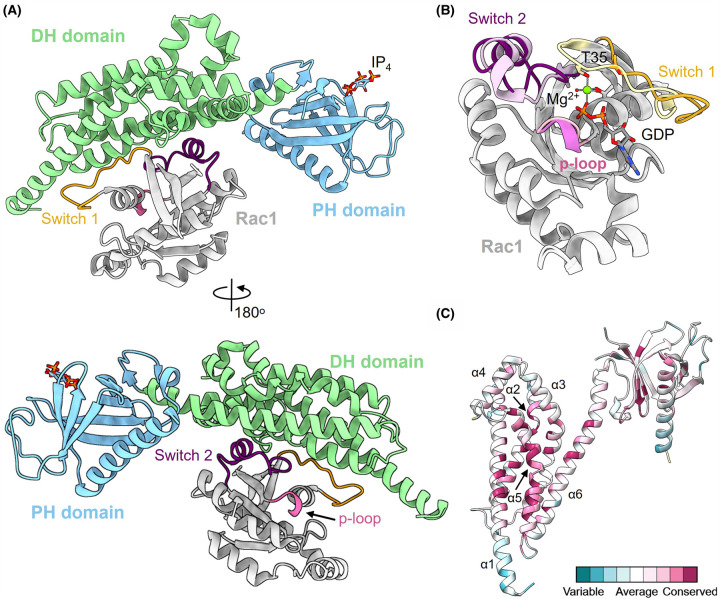
The conserved catalytic P-Rex1 DH-PH tandem domains. (**A**) Structure of the P-Rex1 DH-PH tandem domain in complex with Rac1. The soluble PI(3,4,5)P_3_ analogue inositol (1,3,4,5)-tetrakisphosphate (IP_4_) is modelled in the PH domain binding pocket. (**B**) Superimposition of GEF and GAP bound Rac1 highlighting structural rearrangements of switch regions associated with GDP dissociation. GEF bound switch regions are coloured as in panel a while the GAP bound switch regions are desaturated. (**C**) Consurf [25] analysis of the 71 human Dbl GEF DH-PH domains mapped onto the structure of the P-Rex1 DH-PH tandem. The GTPase binding interface is conserved. PDB ID: 4YON [23], 5D3X [24], 1HE1 [26].

Like many large multi-domain proteins, initial structural insights into P-Rex1 function came from crystal structures of isolated domains rather than the full-length structure. Several structures of the P-Rex1 catalytic DH-PH tandem have been reported in complex with the Rho GTPases Rac1 and Cdc42 [23,24] (Figure 2A). These structures revealed a typical DH domain architecture composed of six α-helices with the sixth α-helix extending to bridge the DH and PH domains. The GTPase binding interface is formed by α-helices 1, 5 and 6 of the DH domain which contact the conserved switch regions of the GTPases. The DH domain promotes structural rearrangement of switch 1 of the GTPases, disrupting the coordination of Mg^2+^ required for GDP binding (Figure 2B). Consequently, GDP dissociates, enabling GTP binding and GTPase activation [9]. This mechanism of Rho GTPase activation is typical of Dbl-GEFs, and accordingly, the DH GTPase interface is highly conserved (Figure 2C) [27–29].

The PH domain does not directly contact the GTPase but functions to bind PI(3,4,5)P_3_ at the membrane, aligning the catalytic DH domain for GTPase interaction [23,24]. The P-Rex1 PH domain comprises a seven-stranded β-sandwich capped by a C-terminal α-helix [23,24]. The structure of the isolated PH domain structures in complex with the soluble PI(3,4,5)P_3_ analogue inositol (1,3,4,5)-tetrakisphosphate (IP_4_) confirm that PI(3,4,5)P_3_ binding occurs to a conserved basic pocket typical for PH domain-containing proteins [24].

## Towards a structural understanding of P-Rex autoinhibition

The catalytic and phosphoinositide binding mechanisms of the P-Rex DH-PH tandem domains are highly conserved across Dbl GEFs. Among the ∼71 human Dbl GEFs, 64 possess the DH-PH tandem domains including the extensively studied Sos1, Tiam1 and Vav1 [30–33]. Of greater mechanistic and structural interest, is the role the remaining five C-terminal domains play in coordinating the strict regulation of P-Rex activity. These regulatory domains function to both lock P-Rex GEFs in the autoinhibited conformation and to co-ordinate their rapid activation downstream of PI(3,4,5)P_3_ production and Gβγ release.

Recombinant full-length P-Rex1 is inactive in *in vitro* GEF activity assays, whereas the DH-PH tandem alone is constitutively active [5]. This observation indicates that the additional domains of P-Rex1 are involved in the autoinhibition of GEF activity, as reported for numerous other GEFs including the eponymous Dbl-GEF [8,34–37]. Several studies have examined the role of specific P-Rex C-terminal regulatory domains in maintaining autoinhibition with the clearest insight derived from two studies examining *in vitro* GEF activity upon a series of C-terminal truncations [5,6,8,10,38–40]. Truncation of P-Rex1 following the DEP1 domain results in a small increase in GEF activity, suggesting that the C-terminal DEP2 through IP4P domains are involved in autoinhibition [8]. However, maximal GEF activity is only observed when the DEP1 domain is also removed [8,38]. The step-wise increase in P-Rex1 GEF activity suggests at least two layers to the P-Rex1 autoinhibitory mechanism. The first layer requires the DEP2 through IP4P domains, while the DEP1 domain facilitates the second layer [8].

**Figure 3.**
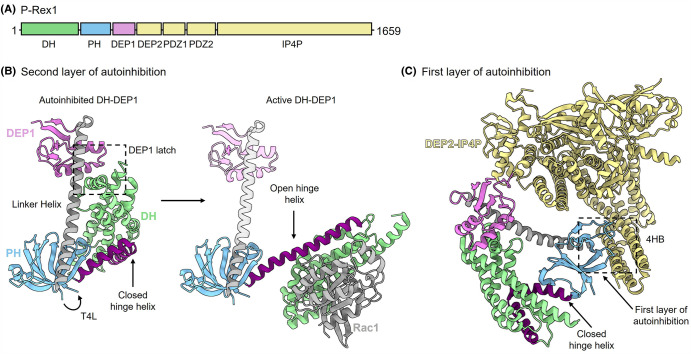
The structural basis of P-Rex1 autoinhibition. (**A**) Domain schematic of P-Rex1. Subsequent structures are coloured according to this schematic. (**B**) Structural basis for the second layer of P-Rex1 autoinhibition. The crystal structure of the P-Rex1 DH-DEP1 domains stabilised by the addition of T4L (position indicated by arrow). The structure adopts a closed autoinhibited conformation as evident by comparison to active DH-PH tandem in complex with Rac1. (**C**) Structural basis for the first layer of P-Rex1 autoinhibition. The full length autoinhibited P-Rex1 cryo-EM model reveals a stabilising interaction between the PH domain and the four α-helix bundle (4HB) region which leads into the IP4P domain. PDB ID: 4YON [23], 7RX9 and 7SYF [8].

The first high-resolution structural insight into P-Rex1 autoinhibition came from the crystal structure of the DH-PH-DEP1 domains stabilised by the addition of T4-lysozyme (T4L) into the unstructured β_3_-β_4_ loop of the PH domain [8] (Figure 3A,B). The DH-PH-DEP1 domains adopt a closed triangular conformation where the catalytic DH domain is buried in a cleft formed by the PH and DEP1 domains. In this closed conformation, the Rac1 binding interface of the DH domain is occluded, inhibiting P-Rex1 GEF activity. This conformation is enabled by the DH-PH domain linker helix adopting a closed helix-turn-helix hinge, allowing the DH domain to interact with the PH and DEP1 domains. Comparison of the autoinhibited structure to the isolated DH-PH tandem domain in complex with Rac1 reveals the DH-PH domain linker α-helix opens by ∼126°, forming a continuous α-helix (Figure 3B) [8,23]. This flexibility is required to expose the DH domain and enable Rac1 binding. Sequence conservation analysis suggests that this autoinhibition mechanism is conserved between P-Rex1 and P-Rex2, with the loss of α-helical propensity at the DH domain hinge site identified in both proteins [8].

Given the flexibility of P-Rex proteins, gaining structural insight into full-length P-Rex1 or P-Rex2 has proved highly challenging. Single-particle cryogenic electron microscopy (cryo-EM) studies of wild-type P-Rex1 suffer from the tendency of the N-terminal domain to denature upon freezing in the required thin layer of vitreous ice [7,8,41]. Nevertheless, the insertion of T4L into the PH domain of full-length P-Rex1 enabled the determination of a 4.5 Å cryo-EM map for the full-length P-Rex1 protein locked in its autoinhibited conformation [8] (Figure 3A,C). Overall, the full-length P-Rex1 structure can be divided into N- and C-terminal lobes that are connected by a flexible linker between the DEP1 and DEP2 domains. The N-terminal lobe comprises the DH-PH-DEP1 domains, which adopt the compact triangular autoinhibited conformation observed in the crystal structure of the isolated domains. The C-terminal DEP2-IP4P domains form a compact structure with extensive contacts between the domains. A four α-helix bundle (4HB) extends from the IP4P domain, contacting the N-terminal PH domain, forming the connection between the N- and C-terminal P-Rex1 lobes [8]. This interface forms the first layer of P-Rex1 autoinhibition by further stabilising the closed conformation of the N-terminal DH-PH-DEP1 catalytic lobe. Recently, a second full-length P-Rex1 cryo-EM structure has been reported [42]. In this P-Rex1 structure, the N-terminal domains are stabilised by the binding of IP_4_ to the PH domain and it is largely analogous to the T4L-fused structure [42].

In summary, the seven P-Rex1 domains co-ordinate to form an autoinhibited closed conformation characterised by two structural layers of autoinhibition. The second layer of inhibition occurs via the 4HB of the IP4P domain contacting the PH domain. The first layer of inhibition occurs via a hinge and latch mechanism where the DEP1 latch stabilises the closed conformation of the hinge helix, thereby occluding the GTPase binding interface of the DH domain.

## Towards uncovering the synergistic mechanism of P-Rex1 activation at the membrane

**Figure 4.**
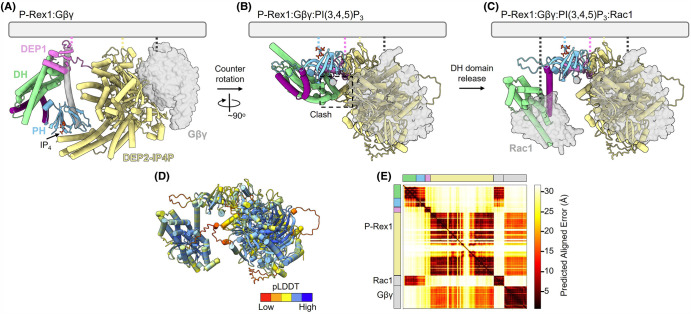
Proposed model of synergistic activation of P-Rex1 by PI(3,4,5)P_3_ and Gβγ. (**A**) Composite model of the autoinhibited P-Rex1:Gβγ complex (PDB ID: 6PCV [7], 7SYF [8]). Dotted lines illustrate the known membrane-binding elements, including loops in the DEP1 domain [38] and the prenylated tail of Gβγ [1]. The DEP2 loops are labelled due to sequence similarity to DEP1, but lipid binding has yet to be investigated. The PI(3,4,5)P_3_ binding pocket, modelled with IP4 (PDB ID: 5D3X [24]), is situated ∼90° off-axis from the other membrane-binding elements. (**B**) Model of the P-Rex1:Gβγ:PI(3,4,5)P_3_ complex demonstrates that counter-rotation of N- and C-terminal lobes of P-Rex1 enables the PH domain to bind PI(3,4,5)P_3_ at the membrane. This rotation is proposed to uncouple the second layer of P-Rex1 autoinhibition, causing a clash between the autoinhibited DH domain and the PH-DEP1 linker helix. The model is a composite of autoinhibited P-Rex1 (PDB ID: 7RX9 [8]) and model in panel (**C**). (**C**) AlphaFold 3.0 [43] model of the active P-Rex1:Gβγ:PI(3,4,5)P_3_:Rac1 complex. A likely steric clash between the DH domain and the PH-DEP1 linker helix, caused by the counter-rotation of the two P-Rex1 lobes, has been proposed to trigger the release of the DEP1 latch [8]. This release is thought to relieve the first layer of autoinhibition and open the DH domain. Subsequently, Rac1 binds to the exposed face of the DH domain. (**D**) AlphaFold 3.0 [43] model as per (**C**) coloured by predicted local distance difference test (pLDDT) and **E**, the AlphaFold 3.0 predicted aligned error plot. The Rac1:DH domain interaction is predicted with high confidence. The Gβγ:PDZ-IP4P interaction is modelled with modest confidence but is consistent with previously published structural data [7]. The relative positions of the N- and C- terminal lobes of P-Rex1 are modelled with low confidence. This is expected because, as in the active conformation, the only contact between the N- and C-termini lobes is the flexible DEP1-DEP2 linker.

Although recent structural insights have uncovered the basis of P-Rex1 autoinhibition, the structure of the active and open P-Rex1:Gβγ:PI(3,4,5)P_3_:Rac1 complex is yet to be determined. As such, it remains to be completely understood how PI(3,4,5)P_3_ and Gβγ synergistically relieve autoinhibition and drive GEF activity. Nevertheless, the autoinhibited P-Rex1 structure, the P-Rex1 DEP2-IP4P:Gβγ complex, and detailed hydrogen-deuterium exchange mass spectrometry (HDX-MS) experiments combine to provide key mechanistic insights into synergistic P-Rex1 activation [7,8,42] (Figure 4).

The first major structural step forward in understanding synergistic P-Rex1 activation came from the cryo-EM structure of the P-Rex1 C-terminal DEP2-IP4P lobe bound to soluble Gβγ [7]. Here, the P-Rex1 C-terminus was revealed to be in an intimate and compact arrangement of the DEP2, PDZ1, and PDZ2 domains around a larger central IP4P domain [7]. Gβγ binds to a conserved P-Rex1 surface, bridging the IP4P and PDZ domains [7]. Interestingly, there is little difference between the C-terminal domains of the autoinhibited P-Rex1 or the P-Rex1 DEP2-IP4P:Gβγ structures. In addition, HDX-MS experiments demonstrated that soluble Gβγ binding does not result in major long-range structural rearrangements of the P-Rex1 autoinhibited domains, which would be required to drive GEF activity allosterically [7,42].

Consistent with these observations, it has long been observed that synergistic activation of P-Rex1 occurs most efficiently downstream of the addition of prenylated-Gβγ and membrane-bound PI(3,4,5)P_3_, rather than soluble Gβγ and soluble PI(3,4,5)P_3_ analogues such as IP_4_ [1,6]. Indeed, while the soluble PI(3,4,5)P_3_ analogue IP_4_ binds within the PH domain binding pocket, it has been reported to inhibit P-Rex1 activity [42]. These results suggest that the membrane is a required third element in P-Rex1 activation and that Gβγ and PI[3,4,5]P_3_ regulate P-Rex protein activity in part by controlling membrane recruitment. This conclusion is further supported by recent HDX-MS experiments demonstrating that, like soluble Gβγ, IP_4_ does not result in any long-range structural rearrangements of autoinhibited P-Rex1 [42].

A potential role of the plasma membrane in directing P-Rex1 activation becomes evident upon inspection of the known membrane binding elements of autoinhibited P-Rex1. P-Rex1 has multiple membrane contact points, including the PH domain PI(3,4,5)P_3_ binding pocket, membrane binding loops in both DEP domains, and the point of prenylation on P-Rex1-bound Gβγ [1,7,24,38]. Interestingly, the PH domain PI(3,4,5)P_3_ binding pocket is off-axis from the other membrane binding regions by ∼90° [8]. Consequently, the simultaneous binding of Gβγ and PI(3,4,5)P_3_ requires significant counter-rotation of the N- and C-terminal lobes around the flexible DEP1-DEP2 linker [8,42]. Such a rotation requires an uncoupling of the PH-4HB interface to unlatch the second layer of autoinhibition. In addition, the first layer of autoinhibition is likely alleviated by a clash between the DH domain and the PH-DEP1 linker helix upon the rotation-induced PH domain movement at the membrane. HDX-MS analysis has provided experimental support for the model, with P-Rex1 binding to PI(3,4,5)P_3_ liposomes initiating deprotection of the P-Rex1 PH-4HB interface and the DH domain [42]. Interestingly, cell-based experiments have indicated that Gβγ may bind to two locations on P-Rex1, providing potential further complexity to the model [40,44]. Studies on P-Rex2b support a second Gβγ binding site in the DH domain, as this variant can bind directly to Gβγ within cells despite lacking the IP4P domain [39]. Further support comes from *in vitro* assays where Gβγ can activate the isolated DH domain and DH-PH tandem domain of P-Rex1 [5,6]. However, direct structural evidence of a second Gβγ binding site within the DH domain has yet to be reported.

While the current model of P-Rex1 activation emphasises the synergistic roles of Gβγ and PI(3,4,5)P_3_, *in vitro* assays demonstrate that Gβγ or PI(3,4,5)P_3_ alone can drive small increases in P-Rex1 GEF activity [1]. HDX data also suggests that PI(3,4,5)P_3_ containing liposomes are sufficient to uncouple both layers of P-Rex autoinhibition, in at least a portion of the molecules present in the system [42]. In addition, in the cellular context, it is often difficult to delineate the requirement for both PI(3,4,5)P_3_ and Gβγ in P-Rex1 activation. For example, while P-Rex1 is readily activated by RTK stimulus alone [14,45–47], it is unclear whether RTK-stimulated PI(3,4,5)P_3_ acts alone or in combination with free Gβγ present at the membrane, in the absence of a direct GPCR stimulus. Moreover, bidirectional cross-talk between GPCRs and RTKs may offer alternative pathways for P-Rex1 activation. For instance, GPCRs can enhance RTK activity to elevate PI(3,4,5)P_3_ levels, and RTKs can stimulate G proteins to release Gβγ subunits [48,49]. Additionally, Gβγ subunits can induce the production of PI(3,4,5)P_3_ by activating PI3K.

Overall, these data suggest that P-Rex1 can sample the open and active conformation when bound to either Gβγ or PI(3,4,5)P_3_. However, the equilibrium shifts further towards the open and active conformation when both Gβγ and PI(3,4,5)P_3_ bind synergistically to induce P-Rex1 counter-rotation at the plasma membrane. Further structural, biophysical, and cellular experiments are required to clearly/definitively delineate the role of each second messenger in order to finalise the model of P-Rex1 synergistic activation.

## Phosphorylation provides further layers of regulation

An additional layer of P-Rex regulation is provided by site-specific phosphorylation, which has been reported to both activate and inhibit P-Rex proteins [15,50–53]. The p21-activated kinases (PAKs), which are activated by GTP bound Rac1, can phosphorylate P-Rex1 following activation of insulin, IGF1 and neuregulin receptors and phosphorylate P-Rex2 following insulin receptor activation [15,54]. The phosphorylation of P-Rex1 by PAKs reduces P-Rex1 association with PI(3,4,5)P_3_ and consequently inhibits GEF activity [54]. PAK-mediated phosphorylation of P-Rex2 reduces GEF activity by inhibiting both PI(3,4,5)P_3_ and Gβγ binding, and membrane association [15]. This suggests that PAKs likely operate via a negative feedback mechanism to terminate Rac1 signalling by inhibiting P-Rex protein membrane association upstream of Rac1 [15,54]. However, the exact locations of PAK phosphorylation of P-Rex proteins are not yet known.

The *known* phosphorylation sites within the P-Rex proteins are primarily within disordered loop regions. For instance, protein kinase A (PKA), whose regulatory domains are known to bind to the P-Rex1 PDZ domains, phosphorylates Ser436 within the membrane binding loop of the P-Rex1 DEP1 domain [38,52]. Phosphorylation of the DEP1 loop leads to decreased P-Rex1 membrane association and subsequent inhibition of P-Rex1 activity [38,52,55]. Conceivably the negative regulation mediated by PAKs on both P-Rex proteins operates through a similar mechanism. The other identified P-Rex phosphorylation sites currently have little mechanistic evidence underpinning their observed function. Phosphorylation sites have been identified in the loop between the DEP2 and PDZ1 domains and within the large loop which extends from the 4HB of the IP4P domain [15,45,46,51,53]. Various combinations of phosphorylation sites have been associated with both increased and decreased Rac1 activation downstream of P-Rex1, however, further investigations are required to probe how the suite of kinases and phosphatases acting upon the P-Rex proteins regulate their activity.

## The P-Rex2:PTEN coinhibitory complex

In comparison to P-Rex1, there is less high-resolution structural data available for P-Rex2. Nevertheless, P-Rex2 is also autoinhibited under basal conditions, and cross-linking mass spectrometry studies indicate that P-Rex2 most likely adopts an autoinhibited conformation comparable to P-Rex1 [2,21]. One striking difference between P-Rex2 and P-Rex1 is the ability of P-Rex2 to bind and form a co-inhibitory complex with PTEN. However, we lack a high-resolution understanding of how P-Rex2 and PTEN form a co-inhibitory complex. It is understood that the formation of the co-inhibitory complex is not dependent on either protein's catalytic activity [11,56]. A series of domain precipitation experiments have mapped the regions required for P-Rex2 PTEN interaction [10–12]. These experiments suggest two interfaces, the first between the C-terminal tail of PTEN and the P-Rex2 IP4P domain, with the second between the PTEN phosphatase domain and the P-Rex2 PH domain [10–12]. A more recent study using surface plasmon resonance and cross-linking mass spectrometry indicated that the insertion of the PDZ interaction motif on the C-terminal tail of PTEN into the binding pocket of the P-Rex2 PDZ2 domain is the primary driver of complex formation [21]. These data led to the proposal of a co-inhibitory model whereby PTEN binding to P-Rex2 blocks the Gβγ binding site of P-Rex2, to inhibit P-Rex2 activation [21]. Simultaneously, it is thought that this interaction occludes the catalytic site of PTEN, inhibiting PTEN activity [21]. In contrast, *in vitro* PTEN assays suggest that the DH-PH tandem is required for PTEN inhibition [10]. Further high-resolution structural studies are required to better understand the molecular details governing P-Rex2 and PTEN co-inhibition.

## Structural insight into cancer-associated P-Rex2 mutations

P-Rex1 overexpression has been identified in melanoma, breast, and prostate cancers [14,45,57,58]. In contrast with P-Rex1, P-Rex2 is frequently mutated in numerous cancers, being the third most mutated protein in studies of primary melanomas, and is highly mutated in pancreatic, breast, and lung cancers [59–63]. Consistent with the role of P-Rex in the regulation of cell survival, proliferation, and migration, their dysregulation in cancer is frequently associated with metastasis and disease progression [14,57,64,65].

**Figure 5.**
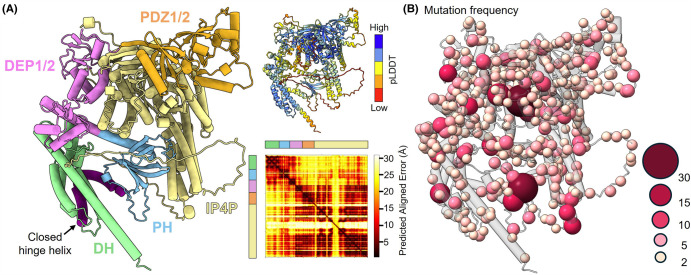
Cancer-associated mutations in P-Rex2 are distributed throughout the protein. (**A**) AlphaFold2 [66–68] model of autoinhibited P-Rex2. Corresponding model coloured by pLDDT with PAE plot. The relative positions of the N- and C-terminal lobes are predicted with moderate confidence, although the PH domain position relative to the C-terminal lobe is predicted with higher confidence. This is likely due to the PH domain 4HB interface and is consistent with the model of P-Rex1 autoinhibition. (**B**) Model of P-Rex2 corresponding to (**A**) with the frequency of cancer associated mutations mapped onto the structure as spheres. Frequency of mutation is depicted by spheres of increasing size and colour intensity. Mutation data was obtained from COSMIC [69]. For clarity, the 231 positions where only a single instance of mutation has been reported are omitted.

Interestingly, the P-Rex2 cancer-associated mutations are distributed throughout the gene and do not cluster to any distinct genetic region. Although the structure of autoinhibited P-Rex2 has not been determined, cross-linking mass spectrometry studies, P-Rex1 homology, and AlphaFold2 modelling [8,21,66,67], allow us to model the position of these mutations throughout the P-Rex2 protein (Figure 5A,B). Similar to analysis at the gene level, the reported mutations are distributed throughout the P-Rex2 protein with no clear mutational hotspots. In fact, of the 1824 cancer-associated SNPs reported in the COSMIC database [69], as of March 2024, only 14 amino acids have been reported as mutated 10 or more times, and these amino acids are distributed throughout P-Rex2. Undoubtedly, a portion of these mutations are passenger mutations and are unlikely to significantly dysregulate P-Rex2 and drive cancer progression. However, this analysis demonstrates that the precise arrangement of all seven P-Rex2 domains is crucial for both autoinhibition and PTEN co-inhibition and that this arrangement may be readily compromised by cancer-associated mutations.

P-Rex mutations after the DH-PH tandem domain that are domain destabilising are likely to both relieve P-Rex2 GEF autoinhibition and PTEN co-inhibitory complex formation, resulting in the potential hyperactivation of Rac GTPase signalling. Consistent with this hypothesis, the three truncation mutations, K278*, E824* and Q1430*, all of which were identified in metastatic melanomas, increase P-Rex2 Rac GEF activity in *in vitro* activity assays [21,56,59]. The truncations also increase xenograft tumour incidence and decrease tumour-free survival time in mice grafted with immortalised human melanocytes [56,59]. The K278* mutation truncates P-Rex2 within the PH domain, while E824* and Q1430* truncate the protein in the IP4P domain, compromising the structure of, or removing, the 4HB. These truncations would remove one or both layers of the P-Rex autoinhibitory mechanism, resulting in increased Rac GEF activity and activation of Rac-mediated cell survival, migration, and invasion pathways.

## Conclusion and future directions

Since the identification and purification of P-Rex1 in 2002 [1], advancements in structural biology techniques over the past 20 years have enabled significant mechanistic insights into the intricacies of P-Rex autoinhibition. This progress has led to the development of a minimal two-step model of P-Rex1 activation at the plasma membrane upon PI(3,4,5)P_3_ and Gβγ binding [8,42]. However, further structural investigations into the open and activated P-Rex:PI(3,4,5)P_3_:Gβγ:GTPase complex are necessary to fully elucidate the mechanistic basis of synergistic P-Rex activation.

Existing structural investigations have predominantly focused on P-Rex1, leaving a gap in the structural data available for the P-Rex2:PTEN co-inhibitory complex, the P-Rex1:PKA complex, and the interaction between P-Rex proteins and mTOR. Currently, the model of P-Rex2:PTEN co-inhibition and the mechanisms triggering the relief of this inhibition are poorly substantiated. While the mechanism of P-Rex1:PKA co-regulation is beginning to be uncovered [38,52,55,70], the structural basis of this interaction is not yet well understood. Lastly, the interaction between P-Rex and mTOR is even less defined. Despite the initial report of P-Rex:mTOR binding in 2007 [22], no further structural evidence of this interaction has been published. With the recent release of structures for both the mTORC1 and mTORC2 complexes [71–73], there is now a solid foundation for structural investigations into P-Rex:mTOR binding and co-regulation.

Given the prevalence of P-Rex dysregulation in metastatic cancers, this intriguing family of GEFs presents promising targets for future therapeutic development. Initial studies have demonstrated the potential tractability of P-Rex proteins as therapeutic targets [74,75]. However, comprehensive therapeutic development programs are required to develop and test specific P-Rex GEF inhibitors across a range of pre-clinical models. A greater understanding of normal P-Rex activity and regulatory networks within the cell will be vital to guide these drug development programs.

## Perspectives

- The P-Rex proteins are large GEFs that serve as a nexus between class 1 PI3K and GPCR signalling pathways. These GEFs integrate signals from both pathways to activate Rac1, which in turn promotes cell growth, survival, and migration.
- Although the regulation of P-Rex proteins is not fully understood, recent breakthroughs in determining multiple structures of autoinhibited P-Rex1 have shed light on the synergistic role of PI(3,4,5)P_3_ and Gβγ in activating P-Rex1. This activation is proposed to occur by relieving a two-step hinge and latch autoinhibitory mechanism. Further research is needed to dissect how each second messenger influences the structural rearrangements required for P-Rex1 activation at the plasma membrane.
- Despite these advances, our understanding of P-Rex2 regulation remains limited. Cancer-associated mutations scattered throughout P-Rex2 emphasise the critical nature of its domain arrangement. However, the unique regulatory mechanism of P-Rex2, involving the formation of a co-inhibitory complex with PTEN, makes it difficult to draw conclusions based on P-Rex1 homology alone. Thus, the regulation of P-Rex2 remains an open question.

## Open Access

Open access for this article was enabled by the participation of Monash University in an all-inclusive *Read & Publish* agreement with Portland Press and the Biochemical Society under a transformative agreement with CAUL.

## Abbreviations

cryo-EM: cryogenic electron microscopy
DEP: Dishevelled EGL10 and pleckstrin
DH: Dbl homology
GEF: guanine nucleotide exchange factor
HDX-MS: hydrogen-deuterium exchange mass spectrometry
mTOR: mechanistic target of rapamycin
PH: pleckstrin homology
PKA: protein kinase A
pLDDT: predicted local distance difference test
